# Thermoacidophilic Bioleaching of Industrial Metallic Steel Waste Product

**DOI:** 10.3389/fmicb.2022.864411

**Published:** 2022-04-13

**Authors:** Denise Kölbl, Alma Memic, Holger Schnideritsch, Dominik Wohlmuth, Gerald Klösch, Mihaela Albu, Gerald Giester, Marek Bujdoš, Tetyana Milojevic

**Affiliations:** ^1^Extremophiles/Space Biochemistry Group, Department of Biophysical Chemistry, University of Vienna, Vienna, Austria; ^2^voestalpine Stahl Donawitz GmbH, Leoben, Austria; ^3^Graz Centre for Electron Microscopy, Graz, Austria; ^4^Department of Mineralogy and Crystallography, University of Vienna, Vienna, Austria; ^5^Faculty of Natural Sciences, Comenius University, Bratislava, Slovakia

**Keywords:** thermoacidophiles, bioleaching, steel waste, archaea, metal recovery

## Abstract

The continuous deposition of hazardous metalliferous wastes derived from industrial steelmaking processes will lead to space shortages while valuable raw metals are being depleted. Currently, these landfilled waste products pose a rich resource for microbial thermoacidophilic bioleaching processes. Six thermoacidophilic archaea (*Sulfolobus metallicus, Sulfolobus acidocaldarius, Metallosphaera hakonensis, Metallosphaera sedula, Acidianus brierleyi*, and *Acidianus manzaensis*) were cultivated on metal waste product derived from a steelmaking process to assess microbial proliferation and bioleaching potential. While all six strains were capable of growth and bioleaching of different elements, *A. manzaensis* outperformed other strains and its bioleaching potential was further studied in detail. The ability of *A. manzaensis* cells to break down and solubilize the mineral matrix of the metal waste product was observed *via* scanning and transmission electron microscopy. Refinement of bioleaching operation parameters shows that changes in pH influence the solubilization of certain elements, which might be considered for element-specific solubilization processes. Slight temperature shifts did not influence the release of metals from the metal waste product, but an increase in dust load in the bioreactors leads to increased element solubilization. The formation of gypsum crystals in course of *A. manzaensis* cultivation on dust was observed and clarified using single-crystal X-ray diffraction analysis. The results obtained from this study highlight the importance of thermoacidophilic archaea for future small-scale as well as large-scale bioleaching operations and metal recycling processes in regard to circular economies and waste management. A thorough understanding of the bioleaching performance of thermoacidophilic archaea facilitates further environmental biotechnological advancements.

## Introduction

The mobilization of metal ions from various insoluble mineral phases has been widely used in biotechnological processes to recover useful metals (White et al., 1998; Donati and Sand, 2007; Khaing et al., 2019). However, the potential of metalliferous steel waste as a circular provider of precious metals for developing technologies has yet to be uncovered. The European Steel Association (EUROFER) states that approximately 278 million tons of steel have been produced in 2020 in Europe (15.2% of worldwide steel production), while generating millions of tons of various waste products (EUROFER, 2020). Many types of hazardous wastes, such as slags, dust, and ash, create space shortages due to their necessary deposition in landfills, contributing to the release of hazardous metalliferous wastes into our environment while leading to a depletion of valuable raw materials (Singh et al., 2013; World Steel Association, 2016).

For this study, a secondary dust waste product of the basic oxygen furnace (BOF) steelmaking process (hereafter referred to as BOF-dust) (Gara and Schrimpf, 1998; voestalpine Stahl Donawitz GmbH, Leoben, Austria) is investigated for microbial metal recovery potential by six thermoacidophilic archaeal strains. The potential of growth, metal solubilization, and the interaction of thermoacidophilic archaea with secondary dust particles was investigated to assess future implementation in high-temperature bioleaching operations. Steel waste products pose a rich resource of metal oxides that need to be recycled or reintroduced into the manufacturing system by natural microbial processes (Pawlowski et al., 1984; Krebs et al., 1997; Barbuta et al., 2015; Waste Disposal Recycling in Steel Industry, 2019). Biomining and bioleaching are well-established operations that are applied in a variety of mineral mining-associated industries and laboratory-scale operations (Bosecker, 1997; Schippers et al., 2013; Jujun et al., 2015; Banerjee et al., 2017; Blazevic et al., 2019). Additionally, microorganisms have been applied to detoxify metal-contaminated compounds derived from industrial processes such as incineration bottom ash, fly ash, slags from smelting processes, heavy metal waste from tanning factories (Batool, 2012; Igiri et al., 2018; Mäkinen et al., 2019; Srichandan et al., 2019), electronic scrap (Brandl et al., 2001), radioactive waste environments (Brim et al., 2003; Manobala et al., 2019), and many more through bioleaching.

Bioleaching is a process based on the ability of microorganisms (archaea, bacteria, fungi, etc.) to convert insoluble metals into a soluble state (Schippers et al., 2013). Usually, this process is performed by acidophilic microorganisms that are genetically equipped to oxidize Fe^2+^ into Fe^3+^ and/or reduced inorganic sulfur compounds (RISCs) at a low pH (acidophilia), preferably by growing in the unity of biofilms and frequently attaching to the mineral material, forming a reaction space (Valdés et al., 2008; Ma et al., 2017; Zhang et al., 2019). Bioleaching at elevated temperatures exploits the potential of thermoacidophilic archaea (~70–80°C), whereas mesophilic bioleaching is performed by bacteria at lower temperatures (~25–40°C) (Norris, 1997). Bioleaching operations executed by thermoacidophilic archaea (e.g., *Sulfolobus* spp., *Metallosphaera* spp., and *Acidianus* spp.) benefit from a more efficient mineral dissolution compared to mesophilic bacterial bioleaching (Marsh and Norris, 1983; Gericke and Pinches, 1999; Wang et al., 2014).

Since bioleaching processes of a large ore mass can generate a significant amount of exothermic heat (Beck, 1967), thermophiles such as *Acidianus brierleyi* proved themselves inherently suitable for high-temperature operations. *A. brierleyi* cells propagate chemolithotrophically by S^0^ or Fe^2+^ redox chemistry (Segerer et al., 1986) and provide a higher sphalerite (CuFeS_2_) leaching rate compared to a mesophilic bacterium *Acidithiobacillus ferrooxidans* culture (Konishi et al., 1998; Saitoh et al., 2017). Furthermore, Sulfolobales representatives, such as *Sulfolobus metallicus* (Huber and Stetter, 1991) and *Sulfolobus acidocaldarius* (Brock et al., 1972), mobilize copper, zinc, and uranium (Gautier et al., 2007), provide desulfurization strategies for coal (Shivvers and Brock, 1973; Kargi and Robinson, 1985; Tobita et al., 1994), and oxidize arsenite to arsenate in culture (Sehlin and Lindström, 1992). Great potential in industrial heap bioleaching is demonstrated by *Metallosphaera hakonensis* (Takayanagi et al., 1996) due to high Fe^2+^ oxidation rates when grown on inorganic sulfur compounds (Bromfield et al., 2011; Shiers et al., 2013). Closely related thermoacidophilic *Metallosphaera sedula* utilizes various metal-containing ores as well as organic carbon sources to feed its respiratory electron transport chain (Huber et al., 1989; Auernik and Kelly, 2010a,b). Besides inherent (chalco-)pyrite bioleaching abilities (Mikkelsen et al., 2007; Ai et al., 2019), *M. sedula* can oxidize solid triuranium octaoxide (U_3_O_8_) to U(VI) while feeding own bioenergetic needs (Mukherjee et al., 2012). Furthermore, *M. sedula* cells are able to biotransform the calcium-tungstate mineral *scheelite* by leaching mineral-bound tungsten into the surrounding, resulting in tungsten carbide layers on the cell surface (Blazevic et al., 2019). Precious metals from synthetic (Kölbl et al., 2017), as well as genuine extraterrestrial materials such as meteorites (ordinary chondrite NWA 1172; Martian breccia NWA 7034) are also solubilized by *M. sedula*, which creates possibilities for future asteroid biomining (Milojevic et al., 2019b, 2021). *A. manzaensis* (Yoshida et al., 2006) is an emerging thermoacidophilic bioleacher, which grows as a facultative anaerobe utilizing molecular hydrogen and elemental sulfur as electron donor under aerobic conditions by using Fe^3+^ as the electron acceptor for anaerobic growth. So far, *A. manzaensis* has merely been investigated for its chalcopyrite leaching efficiency as well as a possible acid mine drainage (AMD) bioremediation agent (Chang-Li et al., 2012; Liu et al., 2016a,b; Nie et al., 2019; Li et al., 2020). Obviously, current and future challenges in circular economy and waste management demand in-depth analyses of microorganisms with great inherent bioleaching potential.

Recent advances in these technologies (e.g., Coetzee et al., 2020; Giachino et al., 2021; Newsome and Falagán, 2021) may contribute to a great extent to the United Nations Sustainable Development Goals (specifically goal number 12), namely, to achieve sustainable and efficient use of natural resources as well as to implement an environmentally sound management of chemicals and all wastes throughout their life cycle by 2030 (United Nations General Assembly, 2015).

In this study, the growth potential of six thermophilic archaeal strains (*S. metallicus, S. acidocaldarius, M. hakonensis, M. sedula, A. brierleyi*, and *A. manzaensis*), their ability to propagate in presence of a multimetallic waste product (BOF-dust), as well as the subsequent solubilization of metals bound to the respective BOF-dust grains is investigated. Furthermore, we focus on the bioleaching potential of *A. manzaensis*, subjecting cells to different leaching parameters (BOF-dust load, pH, and temperature), while investigating the microbial interactions with dust particles *via* scanning and transmission electron microscopy (SEM and TEM) coupled to energy-dispersive spectroscopy (EDS) analysis. In our study, we observe efficient metal solubilization from dust particles by *A. manzaensis* and explore microbial-mineral interactions associated with this case. Our results provide the first insights into laboratory-scale thermoacidophilic bioleaching associated with industrial steelmaking waste products. Integrating not well-studied bioleaching microorganisms into future circular waste management processes might open up a plethora of new applications for environmental biotechnology.

## Materials and Methods

### Strains and Media Composition

*A. manzaensis* (NBRC 100595), *A. brierleyi* (DSMZ 1651), *S. metallicus* (DSMZ 6482), *S. acidocaldarius* (DSMZ 639), *M. hakonensis* (DSMZ 7519), and *M. sedula* (DSMZ 5348) cultures were grown aerobically in their respective media (Table 1). Allen's trace element solution was added to 1 L media, resulting in 1.80 mg MnCl_2_·4H_2_O, 4.50 mg Na_2_B_4_O_7_·10H_2_O, 0.22 mg ZnSO_4_·7H_2_O, 0.05 mg CuCl_2_·2H_2_O, 0.03 mg Na_2_MoO_4_·2H_2_O, 0.03 mg VSO_4_·2H_2_O, and 0.01 mg CoSO_4_ final concentration (for DSMZ 1,651; 6,482; 639; 7,519; 5,348). A solution containing 831 trace elements was added to 1 L media, resulting in a final concentration containing 1 mg ZnSO_4_·7H_2_O, 2 mg CuSO_2_·5H_2_O, 1 mg MnSO_4_·5H_2_O, 0.5 mg Na_2_MoO_4_·2H_2_O, 0.5 mg CoCl_2_·6H_2_O, 1 mg NiCl_2_·6H_2_O, and 5 mg FeSO_4_·7H_2_O (for NBRC 100595). pH was adjusted with 10N H_2_SO_4_ according to the DSMZ/NBRC data_._ Cultivation of thermophilic archaea was performed as described earlier (Milojevic et al., 2019b) in 1 L modified Schott-bottle bioreactors (Duran DWK Life Sciences GmbH, Wertheim/Main, Germany), equipped with a thermocouple connected to a heating and magnetic stirring plate (RH basic 2 IKAMAG, IKA-Werke GmbH & Co. KG, Staufen, Germany) for temperature and agitation control (Supplementary Figure S1). Each bioreactor was equipped with three 10 ml graduated pipettes, permitting carbon dioxide and air gassing (gas flow rates 0.5 NL/min, respectively, MFC 8741, Bürkert GmbH & Co. KG, Ingelfingen, Germany) and sampling of culture. The graduated pipettes used for gassing were connected by silicon tubing to sterile 0.2 μm filters (Millex-FG Vent filter unit, Merck KGaA, Darmstadt, Germany). Graduated pipettes used for sampling were equipped with a Luer-lock system to permit sampling with sterile syringes (Soft-Ject, Henke Sass Wolf, Tuttlingen, Germany). Off-gas was forced to exit *via* a water-cooled condenser (Carl Roth GmbH & Co. KG, Karlsruhe, Germany). Respective temperatures inside the bioreactors were controlled by electronic thermocouple *via* heating and magnetic stirring plates. For chemolithotrophic growth, cultures were supplemented with 10 g/L BOF-dust. Before addition to the microbiological medium, BOF-dust samples were heat-sterilized at 180°C in a laboratory oven for a minimum of 24 h prior to autoclavation at 121°C for 20 min. Cell growth was examined *via* cell counting (Neubauer Chamber, Carl Roth GmbH & Co. KG, Karlsruhe, Germany) and the release of soluble metals into the culture medium.

**Table 1:** Detailed microbiological media composition for the six thermoacidophilic strains.

**[g]**	***A. manzaensis***	***A. bierleyi***	***M. sedula***	***M. hakonensis***	***S. metallicus***	***S. acidocaldarius***
(NH_4_)_2_SO_4_	0.132	3.00	1.30	1.30	1.30	1.30
KH_2_PO_4_	0.041	0.50	0.28	0.28	0.28	0.28
MgSO_4_·7H_2_O	0.49	0.50	0.25	0.25	0.25	0.25
CaCl_2_·2H_2_O	0.009	-	0.07	0.07	0.07	0.07
FeCl_3_·6H_2_O	-	-	0.02	0.02	0.02	0.02
KCl	0.052	0.10				
Ca(NO_3_)_2_	-	0.01				
Yeast extract	-	-	-	-	-	0.01 % (w/v)

### Metal Release Analysis (ICP-MS)

To determine the extracellular concentrations of metal ions mobilized from the metal-containing waste product, samples of thermoacidophilic cultures were clarified by centrifugation. Cells were harvested at the early stationary growth phase for inductively coupled plasma (ICP) measurements. Samples of the resulting supernatants were filtered (0.44 μm pore size, Macherey-Nagel GmbH & Co. KG, Germany). A total of 18 elements (Fe, Cu, Ni, Zn, Cr, Cd, Mn, Ca, Mg, Al, Co, V, Ti, Sr, Pb, Mo, Ba, As) were analyzed by ICP mass spectrometry (ICP-MS). Abiotic samples (containing BOF-dust and respective microbiological media but no cells) and zero time points (at start of cultivation) were analyzed in each experiment, and their corresponding values are already subtracted in all figures presented in this study.

### Wavelength-Dispersive X-Ray Fluorescence Analysis (WD-XRF)

The BOF-dust sample used in the study was analyzed using wavelength-dispersive X-ray fluorescence (WD-XRF) spectrometer Zetium PW 5400, Panalytical and SuperQ-program. The evaluation of the measuring data was done using Uniquant program, hence the chemical composition was ascertained. In parallel, the loss of ignition of the sample was determined at 1,000°C. The results of the evaluation of the bulk elemental composition of BOF-dust *via* WD-XRF analysis are presented in Supplementary Table S1.

### Scanning and Transmission Electron Microscopy

For scanning transmission electron microscopy coupled to energy-dispersive X-ray spectroscopy (SEM-EDS) analysis*, A. manzaensis* cells were collected, primary fixed with 2.5% glutaraldehyde (in 1 M Na-cacodylate buffer), and post-fixed for 2 h in 1% osmium tetroxide (OsO_4_). Subsequently, samples were washed (1.5 × PHEM-buffer) and dehydrated by a gradual ethanol series (30, 50, 70, 80, 90, 95%, abs.) and acetone (100%). Samples were dried by critical point drying (Critical Point Dryer, Leica EM CPD300, Leica Microsystems GmbH, Wetzlar, Germany), gold sputtered (JEOL JFC-2300HR from Jeol GmbH, Freising, Germany), and analyzed *via* JEOL IT300 Microscope at 20 kV acceleration voltage (Jeol GmbH, Freising, Germany). EDAX Detector (AMETER Material Analysis Division, Ametek GmbH, Weiterstadt, Germany) and energy-Dispersive X-ray spectroscope (EDX) were used for the elemental analysis at an acceleration voltage of 25 kV. Spectra and mappings with the elements of chosen areas were determined using TEAM™ EDS Analysis System (Texture and Elemental Analytical Microscopy; detector Octane Plus) program.

Sample preparation for STEM-EDS has been performed by focused ion beam (FIB) sputtering using a FEI Quanta 3D FEG instrument, equipped with an electron column hosting a field-emission electron source and an ion column hosting a Ga-liquid metal ion source (LMIS) as described earlier (Milojevic et al., 2021). Before sputtering, a Pt layer was deposited on the sample surface as a protective layer by FIB Pt deposition at 16 kV IB acceleration voltage. STEM and energy-dispersive X-ray spectrum (EDS) images were acquired from *A. manzaensis* cultures grown on BOF-dust. Elemental maps and spectra were processed with Gatan Digital Micrograph software (Gatan Inc.). Element quantification for EDS spectra was performed by using the k-factor method.

### Crystal X-Ray Diffraction

The crystalline phase was collected from cultures of *A. manzaensis* grown on BOF-dust. Single crystals were mounted on loops and examined using a Bruker D8 Venture, operated with multilayer monochromator, INCOATEC microfocus sealed tube (λ (MoKα) = 0.71073 Å) and CMOS Photon Detector.

## Results

### Microbial Growth on BOF-Dust

**Figure 1:**
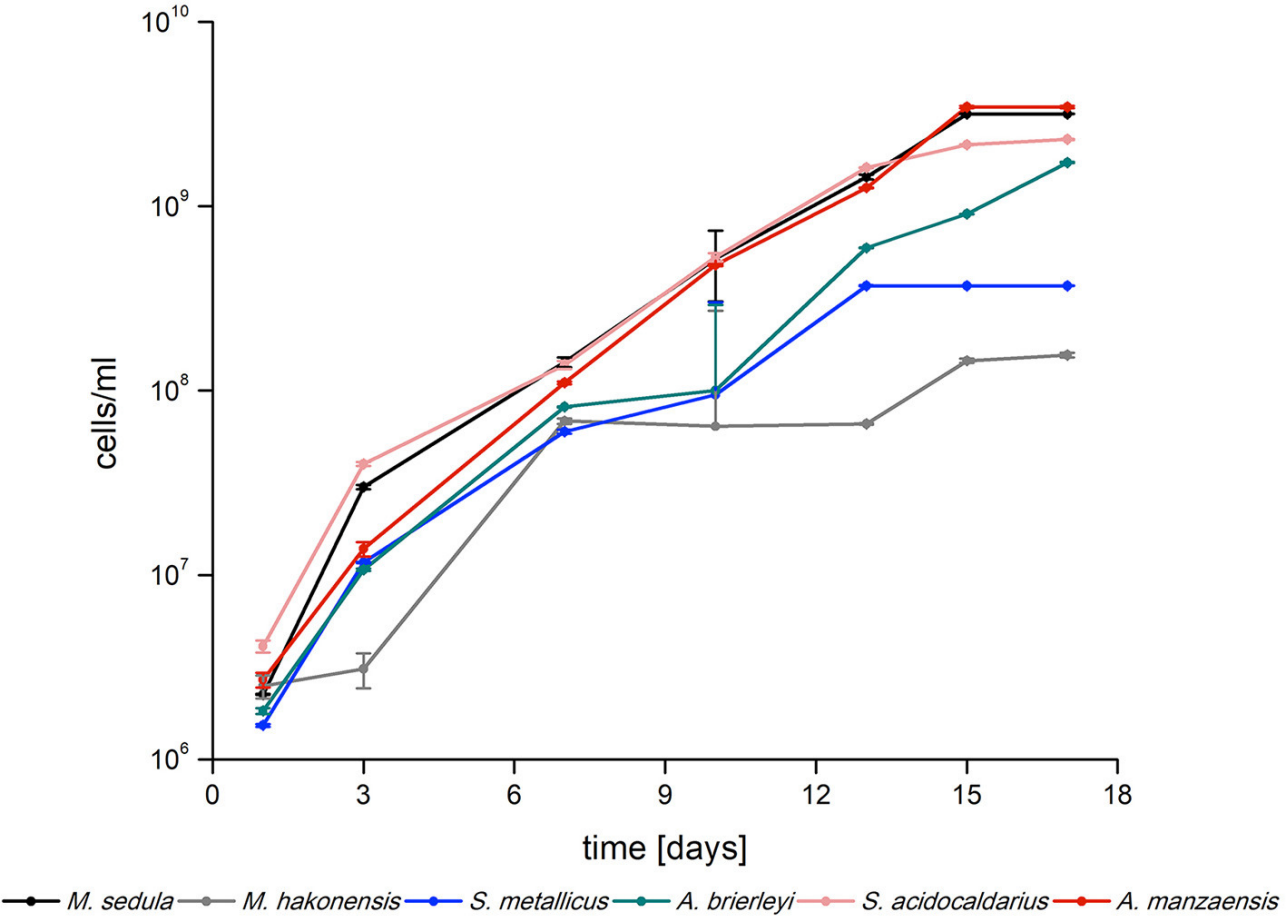
Logarithmic growth curves of *Sulfolobus* spp., *Metallosphaera* spp., and *Acidianus* spp. grown on BOF-dust (10 g/L) over time (*n* = 3).

Six archaeal strains of the Sulfolobales order (*A. manzaensis, A. brierleyi, S. metallicus, S. acidocaldarius, M. hakonensis*, and *M. sedula)* were grown in their respective media (Table 1, also refer to the “Materials and methods” section) supplemented with BOF-dust to examine growth capacity and culture maintenance in conditions of a high metal content. In course of 17 days of cultivation supplemented with CO_2_-gassing and BOF-dust (single portion, 10 g/L culture), microbial growth was monitored by phase-contrast/epifluorescence microscopy and metal release. Each culture was inoculated with approximately 10^6^ cells/ml and during 17 days of operation time, growth of all six thermoacidophiles on BOF-dust became apparent through a 100- or 1,000-fold increase in cell densities at the final point of cultivation (Figure 1). *A. manzaensis* and *M. sedula* were among the highest proliferating strains with final densities of 3.5 × 10^9^ and 3.2 × 10^9^ cells/ml, respectively, whereas *M. hakonensis* and *S. metallicus* exhibited lowest cell densities of all six strains after 17 days of cultivation (maximum densities 1.6 × 10^8^ and 3.7 × 10^8^ cells/ml, respectively). *S. acidocaldarius* and *A. brierleyi* reached cell numbers (2.3 × 10^9^ and 1.7 × 10^9^ cell/ml, respectively) comparable to *A. manzaensis* and *M. sedula*.

### Metal Release of Six Thermoacidophilic Archaea

**Figure 2:**
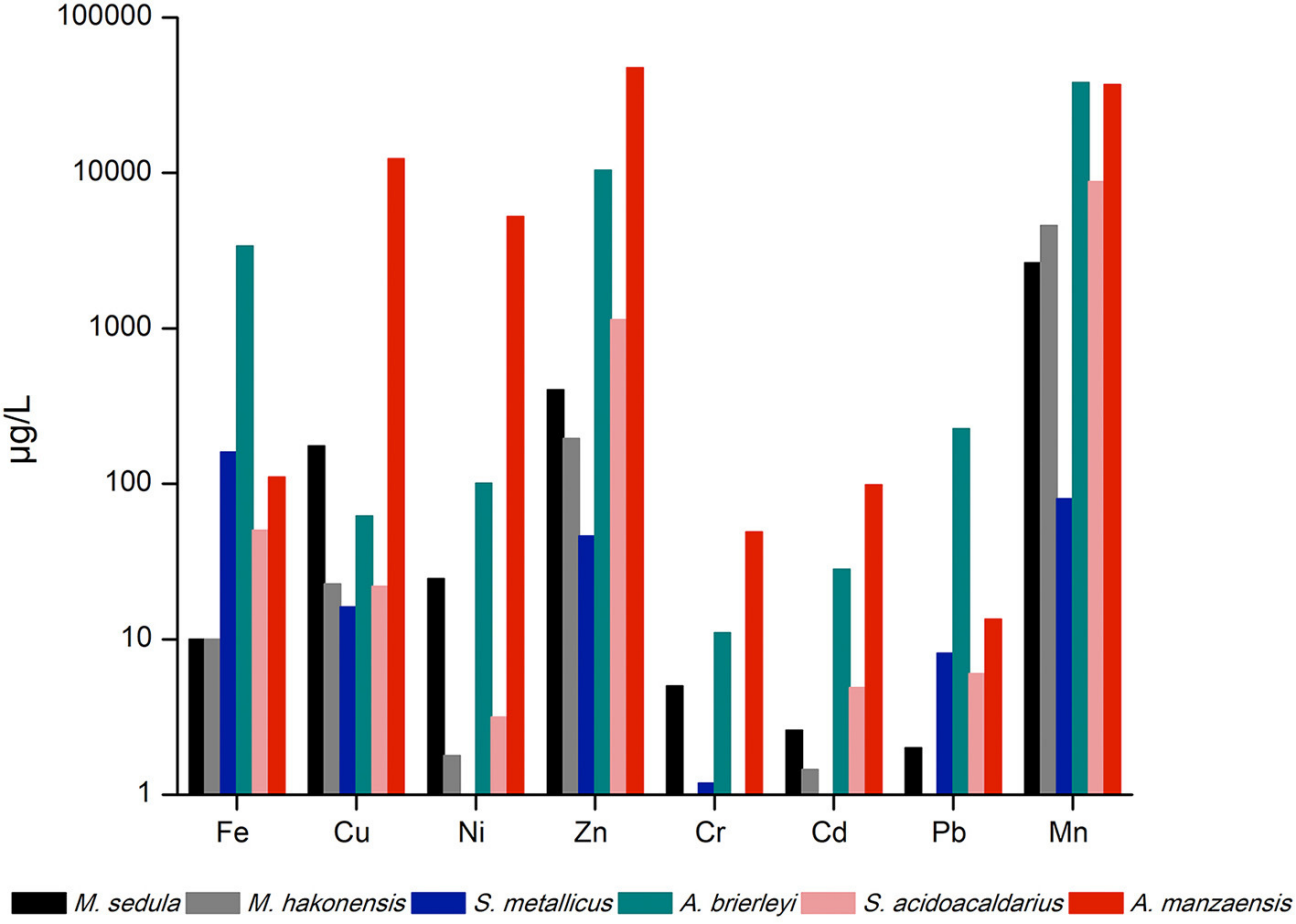
ICP-MS analysis of released metal ions in the supernatant of thermoacidophilic cultures grown on BOF-dust (10 g/L).

The six aforementioned thermoacidophilic strains were tested for their potential to mobilize metals from BOF-dust particles into their respective medium (leachate solution). A comparative analysis of leached elements among thermophiles was carried out *via* ICP-MS. The most abundant elements in the microbial leachate of *A. manzaensis* were Mn, Cd, Cr, Zn, Ni, and Cu, whereas Fe, Pb, and Mn were released more efficiently by *A. brierleyi. M. hakonensis* was able to mobilize Mn, Zn, Cu, Fe, Cd, and Ni from the dust product, but no release of Cr or Pb could be detected. Similar to the absence of solubilized Cr in the *M. hakonensis* culture, *S. acidocaldarius* did not leach any Cr into the microbiological medium. Yet, the overall bioleaching performance of *S. acidocaldarius* is comparable to *M. sedula* and *S. metallicus*, with *S. acidocaldarius* solubilized more Mn, Zn, and Fe into the leachate solution than *M. sedula* and *S. metallicus*. Both Ni and Cd were not detected in the *S. metallicus* culture (Figure 2).

### *A. manzaensis*-Mediated Bioleaching Under Various Conditions

**Figure 3:**
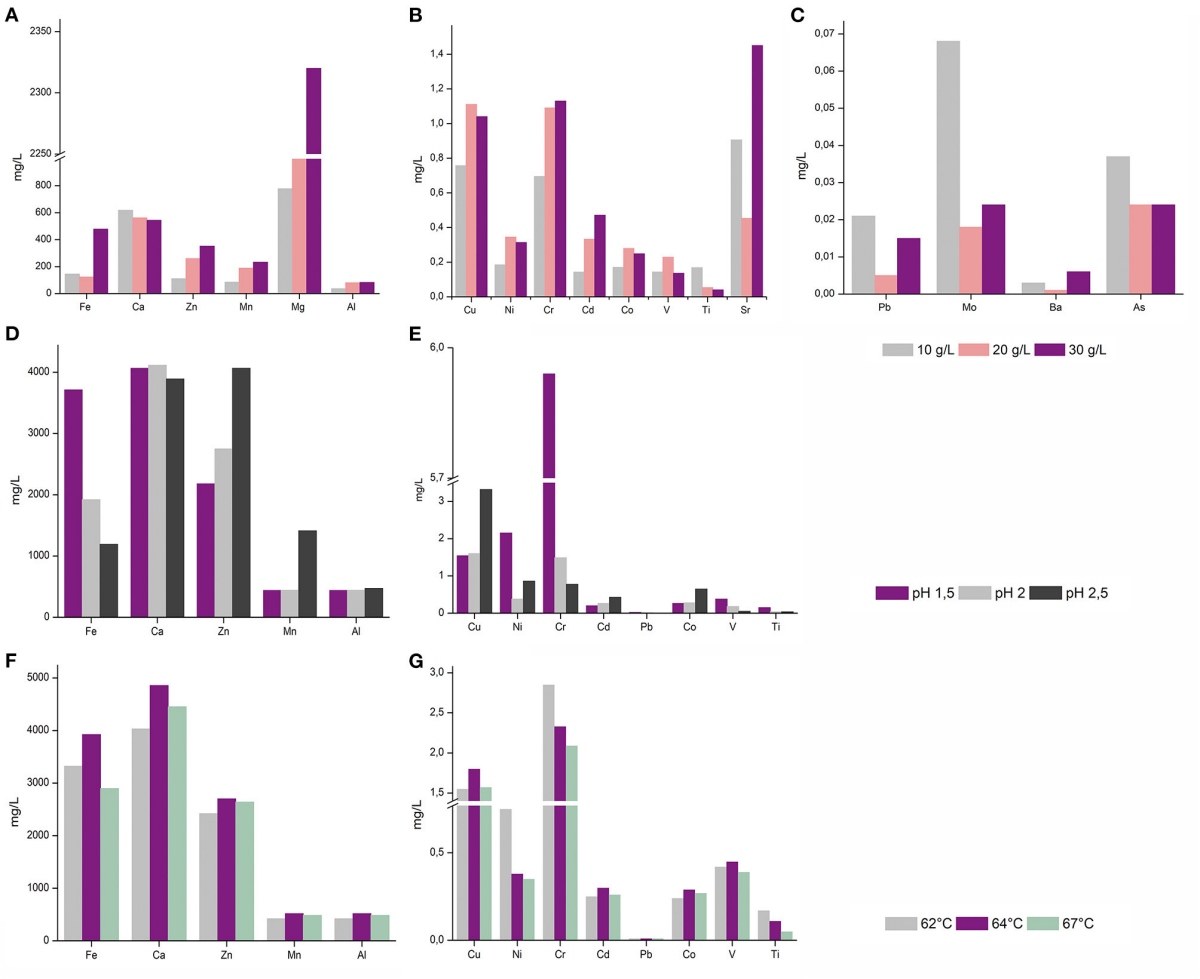
ICP-MS analysis of released metal ions in leachate solution of *A. manzaensis* grown on BOF-dust at different parameters. **(A–C)** *A. manzaensis* grown on increasing amounts of dust (10/20/30 g/L); **(D,E)** *A. manzaensis* grown at different pH values (1.5/2.0/2.5); **(F,G)** *A. manzaensis* grown at different temperatures (62°C/64°C/67°C).

*A. manzaensis* cells were subjected to further in-depth experiments due to the overall high bioleaching performance and the most outstanding Cr leaching abilities compared to other strains tested. To assess cultivation parameters for more efficient *A. manzaensis* bioleaching operations, parameters such as BOF-dust load, operation pH and temperature were examined. For future applications in bioleaching with this archaeon, exposure to higher concentrations of the multi-metallic waste product is favorable. Therefore, *A. manzaensis* cultures were fed with increasing BOF-dust concentrations (10/20/30 g/L) (Figures 3A–C), and subsequent metal release was investigated *via* ICP-MS. Elements, such as Fe, Zn, Mn, Mg, Cr, Cd, and Sr, were detected at elevated levels respective to increasing dust concentrations, while the release of Ca, Al, Ni, Co, V, Ti, Pb, Mo, and As did not correspond to increasing concentrations of dust in the cultures (Figure 3A). For aerobic growth, *A. manzaensis* tolerates pH ranges from 1.0 to 5.0 with the optimum being around 1.2–2.0 (Yoshida et al., 2006). ICP-MS analysis for varying pH values set at 1.5, 2.0, and 2.5 points out that in a more acidic environment of pH 1.5 *A. manzaensis* cells mobilize Fe, Ni, V, Ti, and most notably Cr with the highest efficiency from dust particles into solution (Figures 3D,E). In contrast, a higher pH at 2.5 supports Zn, Mn, Cu, Cd, and Co bioleaching, while a neglectable change in metal mobilizing performance for all elements at pH 2.0 became apparent. Regarding operation temperature, *A. manzaensis* cells are able to grow in a temperature range of 60–90°C (Yoshida et al., 2006), in previous bioleaching operations as well as for this study, *A. manzaensis* was cultivated at 65°C (Liu et al., 2016a,b; Nie et al., 2019). Slight temperature shifts (62°C/64°C/67°C) did not result in a significant change in bioleaching performance (Figures 3F,G). An operating temperature of 64°C yields slightly higher concentrations of Fe, Ca, Zn, Mn, Al, Cu, Cd, Co, and V, even though these differences are not substantial. Interestingly, the most efficient Cr leaching was achieved at 62°C, and the least efficient temperature was 67°C.

### SEM Observations of Raw and Bioprocessed BOF-Dust Particles

**Figure 4:**
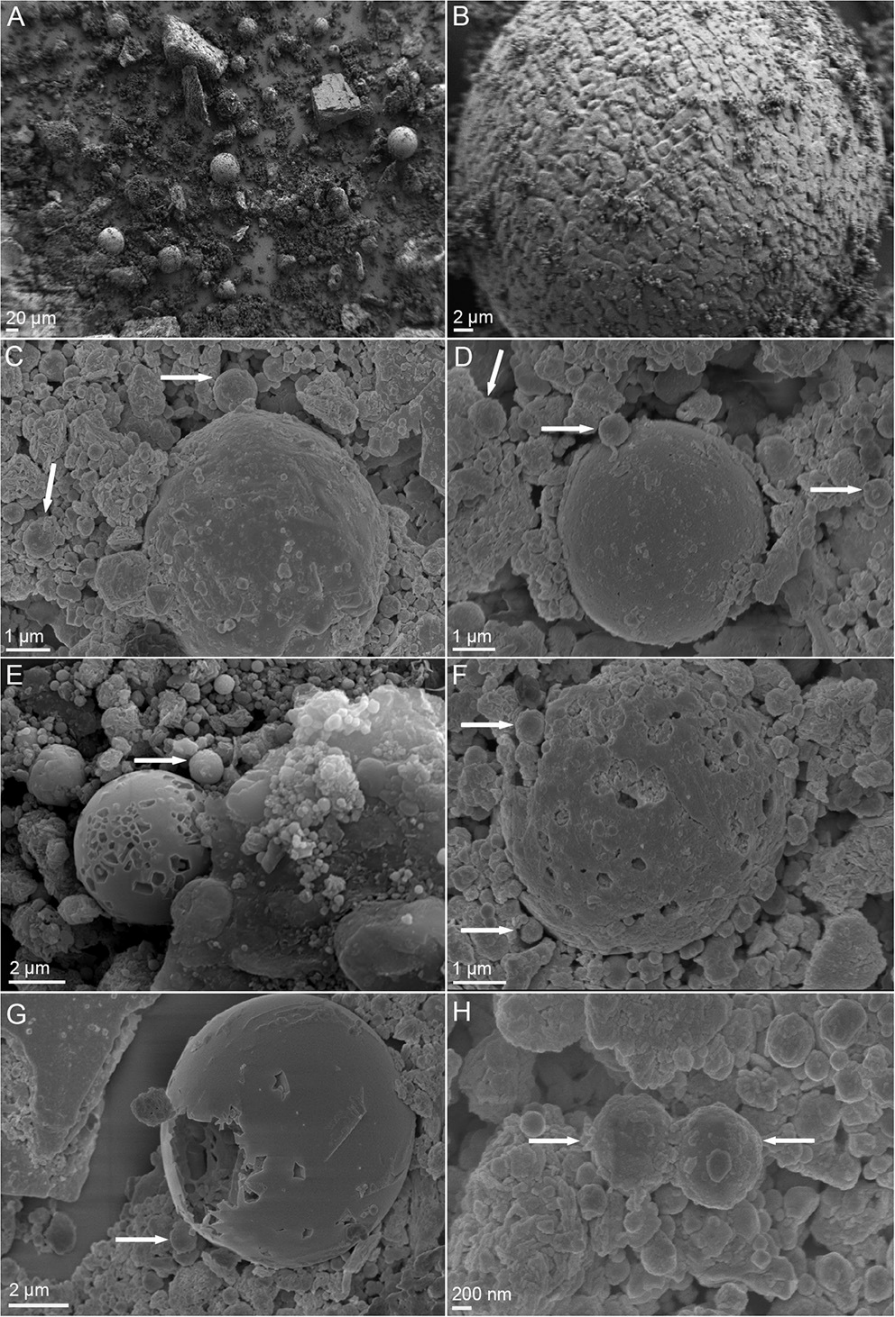
Scanning electron microscopy (SEM) images of BOF-dust particles and cells of *A. manzaensis*. **(A)** Overview of raw, unprocessed dust sample; **(B)** Enlarged image of unprocessed dust corn particle; **(C,D)** Microbially processed sample, shown are dust corn particles with co-localized *A. manzaensis* cells. **(E–G)** Microbially processed sample, shown are dust corn particles with co-localized *A. manzaensis* cells; **(H)** Microbially processed sample, shown are dividing cells of *A. manzaensis*. White arrows indicate cells (~1 μm size). Microbially processed grains of waste dust product are bigger (up to 5–20 μm) and are shown in **(C–G)**, revealing their microbially etched surface.

**Figure 5:**
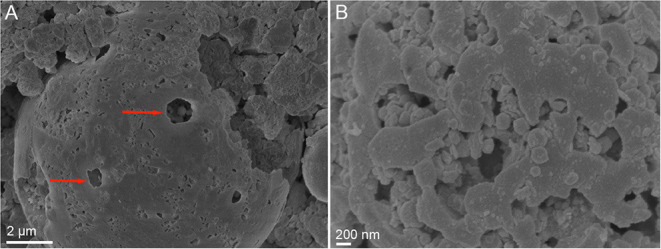
SEM images showing the microbially etched surface of grains of waste material product bioprocessed by *A. manzaensis*. **(A)** The microbially etched pits are depicted by the red arrows. **(B)** Magnified SEM image of microbially etched surface.

To further investigate the interactions of *A. manzaensis* cells with BOF-dust particles, SEM was applied on unprocessed (raw, not incubated with microbial cells). BOF-dust samples as well as with BOF-dust samples after cultivation with *A. manzaensis*. SEM examination of unprocessed dust particles demonstrates that big, spherical grains with a diameter ranging from 2 to 80 μm exist among significantly smaller and finer dust grains (Figure 4A). A close-up SEM caption of an approximately 50-μmsized dust grain exhibits a rough and rippled surface, covered in dust debris (Figure 4B). After a total of 14 days of cultivation of *A. manzaensis* supplemented with 10g/L BOF-dust, observations from SEM analysis show overall small dust particles approximately 2–5 μm with various surface textures and structures (Figures 4C–G). The rough and rippled surface of unprocessed particles (Figure 4B) transformed into extremely smooth-looking structures or structures with porous or broken surface (etched) upon 14 days of cultivation (Figures 4C–G, Figure 5; Supplementary Figures S2,S3). Additionally, *A. manzaensis* cells appear as regular cocci that are about 0.6–1.0 μm in size, co-localizing with BOF-dust particles (Figures 4C–H). In Figures 4C–G, coccoid cells of *A. manzaensis* are shown attached or close to single grains of waste dust material (>5 μm), which have a microbially etched surface. The exemplary biotransformed grains of waste dust material are shown in Figures 4C–G, Supplementary Figures S2, S3. The magnified SEM images (Figure 5, Supplementary Figure S3B) illustrate a close-up of grains of waste product material bioprocessed by *A. manzaensis*. The red arrows in Figure 5A show exemplary microbially etched pits on the grain surface. Single etched pits can be bigger than 2 μm (Figure 4G). A cell division of *A. manzaensis* (Figure 4H) and cell attachment to a big grain of waste dust material (Figure 4D) are depicted by the white arrows. Overall, the dust grains are covered by multiple etched pits upon cultivation with *A. manzaensis*, indicating the attack of microbial cells that use the metal-bearing waste product material as a nutrient and as the energy source. Furthermore, small and round particles (around 200 nm) are highly abundant in all biogenic samples (Figures 4, 5, Supplementary Figures S2, S3), but absent in unprocessed BOF-dust. These round-shaped nanoparticles can represent well-known extracellular vesicles that are secreted by thermoacidophilic archaea of the Sulfolobales order to catalyze metal oxidation and facilitate mineral solubilization under the energy-starving chemolithoautotrophic conditions (Johnson et al., 2018). The exemplary microbially colonized BOF-dust gains that are embedded in the network of cells and their vesicles are shown in Supplementary Figures S2C,D. We have also previously observed the formation of such vesicles during growth on other multimetallic materials (Milojevic et al., 2019a,b, 2021).

### Analytical Spectroscopy Analysis of Microbial-Metal Interface

**Figure 6:**
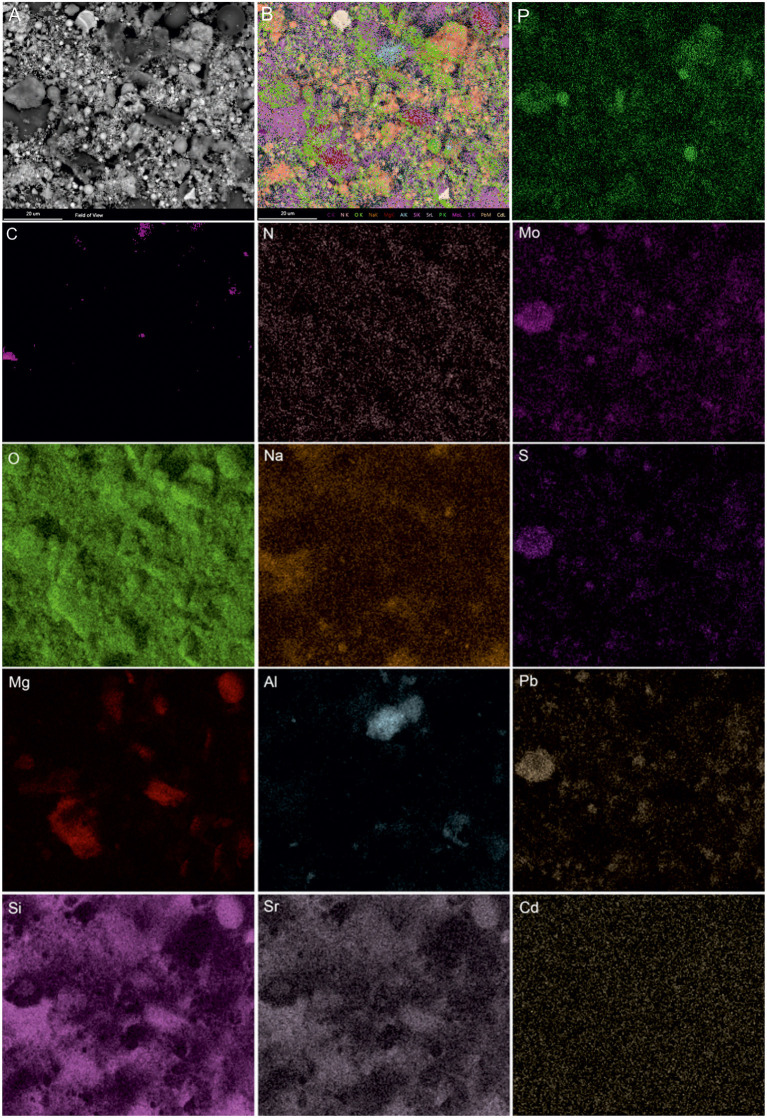
SEM-EDS investigations of microbial-mineral assemblages formed in cultures of *A. manzaensis* grown on BOF-dust. Shown are the SEM image of a microbially processed sample used for the EDS spectrum image acquisition, composite image, and corresponding elemental distribution maps of phosphorus (P), carbon (C), nitrogen (N), molybdenum (Mo), oxygen (O), sodium (Na), sulfur (S), magnesium (Mg), aluminum (Al), lead (Pb), silicon (Si), strontium (Sr), and cadmium (Cd).

**Figure 7:**
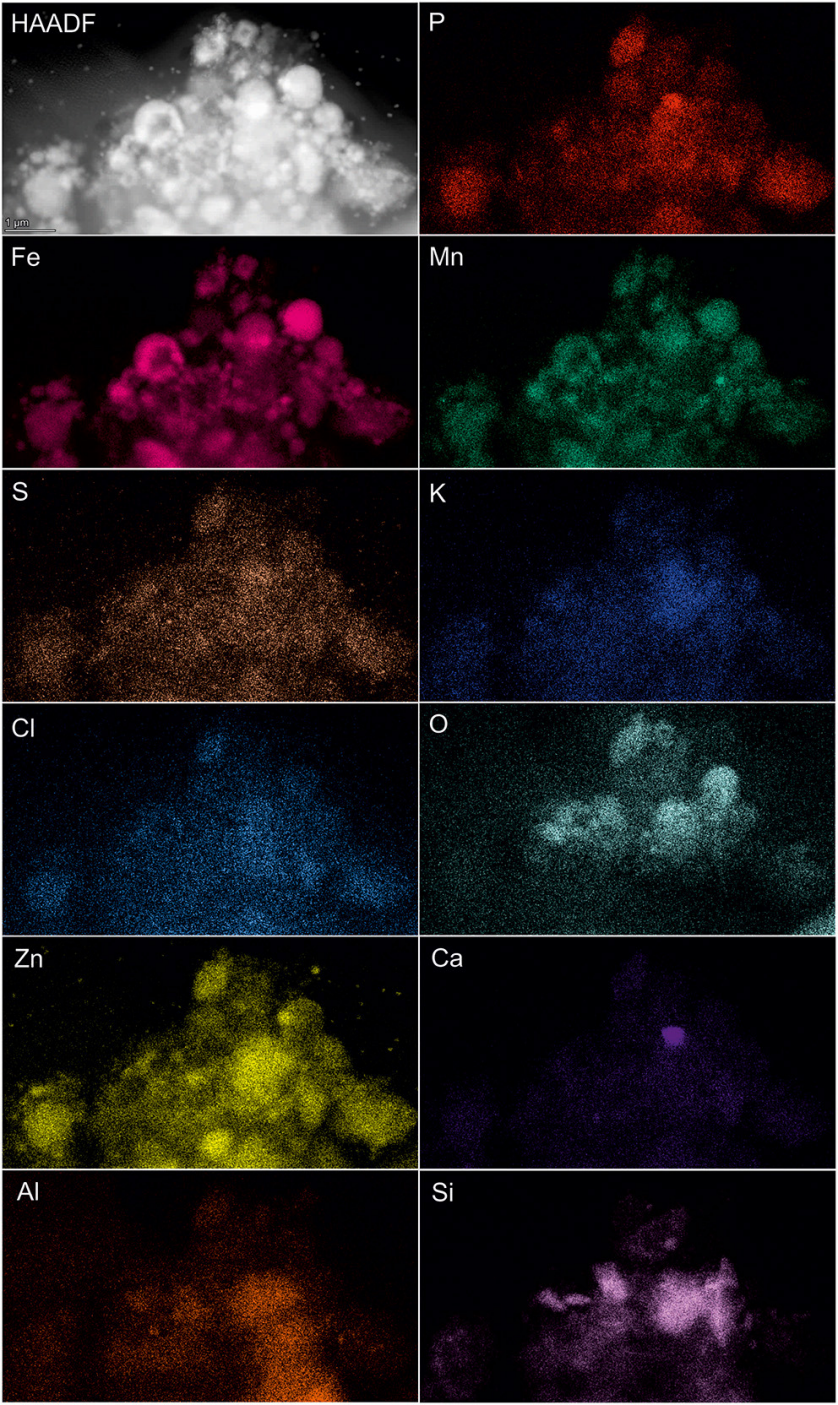
Elemental ultrastructural analysis of microbial-mineral assemblages formed in cultures of *A. manzaensis* grown on BOF-dust. HAADF-STEM image of a microbially processed sample used for the EDS spectrum image acquisition and corresponding elemental distribution maps of phosphorus (P), iron (Fe), manganese (Mn), sulfur (S), potassium (K), chlorine (Cl), oxygen (O), zink (Zn), calcium (Ca), aluminum (Al), carbon (C), nitrogen (N), and silicon (Si).

SEM-EDS was applied to localize element distribution among highly heterogeneous microbial-mineral content of *A. manzaensis* cultures grown on BOF-dust. Elemental EDS maps detected an abundance of Fe, S, P, Co, Pb, and Mo, which are distributed evenly among the microbially processed sample after 14 days of cultivation, whereas Cr, Mn, Al, and Mg were detected in specific spots of the same sample (Figure 6). For further investigations at high resolution, a thin section was produced *via* a FIB milling and examined in STEM mode. The STEM-EDS results demonstrate the content and distribution of metals and metalloids in microbial-mineral assemblages (Figure 7). The elemental maps show that microbial-mineral assemblages are enriched with P, Fe, Mn, Zn, and O. Biologically important elements such as S, K, and Cl are evenly distributed within a microbial-mineral assemblage. Additionally, Si and Al deposits are detected in the sample.

**Figure 8:**
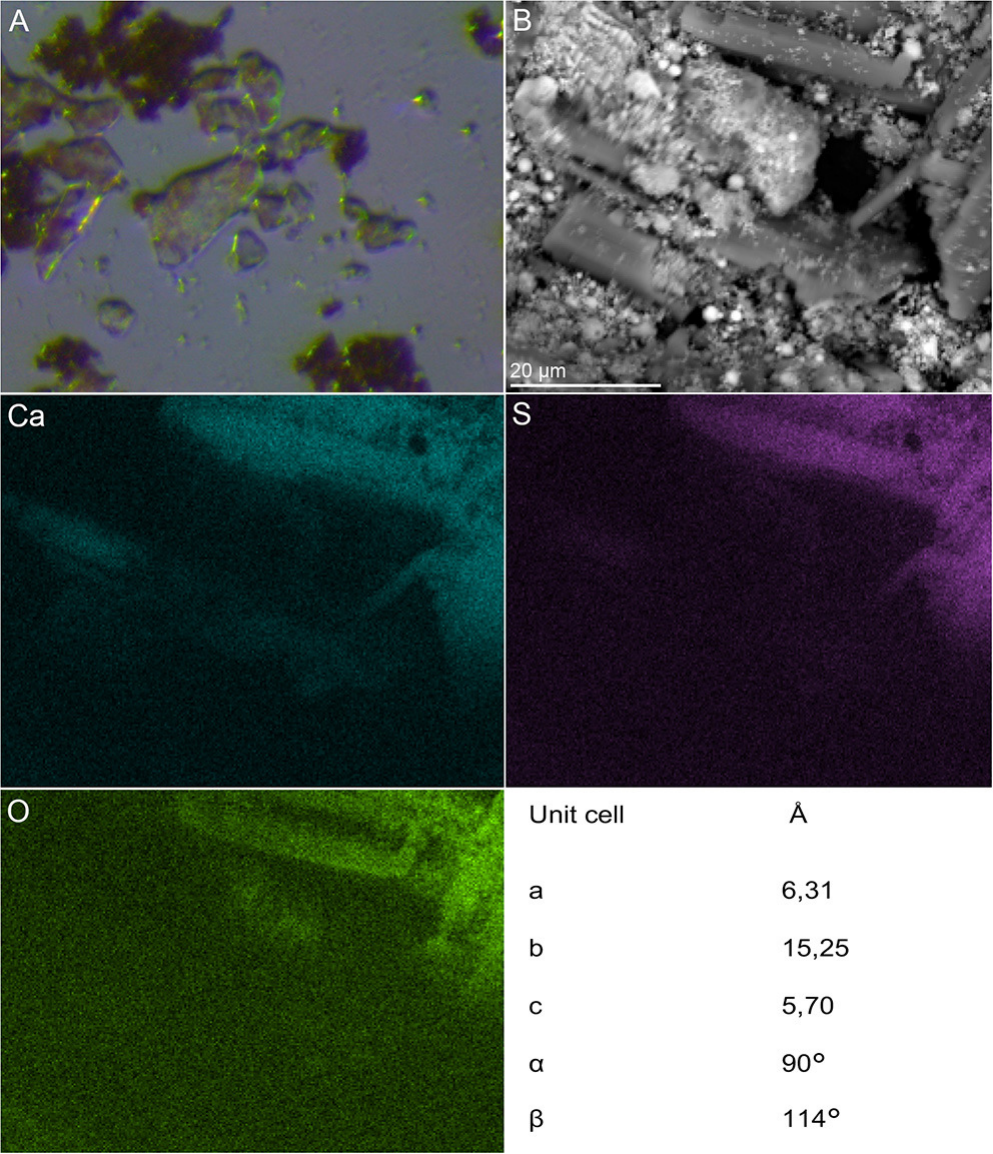
Microscopic and spectroscopic analysis of gypsum crystals detected in *A. manzaensis* culture supplemented with BOF-dust. **(A)** Light microscopy of gypsum crystals in active culture. **(B)** SEM image of bioprocessed BOF-dust sample used for the EDS spectrum image acquisition and corresponding extracted calcium (Ca), sulfur (S), and oxygen (O) elemental maps with unit cell parameters detected with single-crystal X-ray diffraction (XRD) analysis.

Moreover, the formation of crystalline material (gypsum (CaSO_4_ × 2H_2_O) crystals) was observed in *A. manzaensis* cultures after 7 days of cultivation on BOF-dust. No crystals were detected in corresponding abiotic controls that comprise only BOF-dust and microbiological medium. Gypsum crystals were initially observed in course of light microscopy investigations (Figure 8A), followed by SEM-EDS analysis (Figure 8B and elemental maps), and subsequently analyzed *via* single-crystal X-ray diffraction (XRD) to determine the structural properties and nature of the crystals. The obtained unit cell parameters (a = 6.31Å; b = 15,25Å; c = 5,70Å; α = 90°; β = 114°) were identified as calcium sulfate dihydrate (CaSO_4_ × 2H_2_O) (Figure 8), indicating the occurrence of calcium sulfate in the bioleachate solution. SEM-EDS investigation of putative gypsum crystals in the microbial culture corresponds to light microscopy and XRD unit cell parameters (Figure 8).

## Discussion

All six thermoacidophilic archaeal strains cultivated for this study were able to grow chemolithotrophically on a high metal-content BOF-dust that is generated during the BOF steel production processes. Our cultivation parameters (strain-specific medium, temperature, acidity) were selected according to commonly applied cultivation parameters for each strain (refer to the “Materials and methods” section). All of the cultivated strains were able to proliferate successfully in course of 17 days. Inherent resistance to a (heavy) metalliferous environment was one of the criteria for strain selection. Previous extensive work with *M. sedula* in mineral- and metal-containing environments made this archaeon a prime candidate (Auernik and Kelly, 2010b; Blazevic et al., 2019; Milojevic et al., 2019b, 2021), along with its close relative *M. hakonensis*. Interestingly, a comparison of metal solubilization capacities of *M. hakonensis* and *M*. *sedula* grown on BOF-dust-supplemented medium favors *M. sedula*, which was able to extract Cr and Pb from BOF-dust grains (Figure 2). Silica content in BOF-dust (Supplementary Table S1) may be solubilized chemically by the acidic pH of the surrounding medium and is subsequently precipitated onto *M. hakonensis* cells during bioleaching operation (Usher et al., 2009). Under these circumstances, a fusion barrier can be created that consequently slows down the release of metals from dust grains (Usher et al., 2009). This phenomenon has already been observed for *M. hakonensis* in heap bioleaching operations and might be applicable to other thermoacidophilic strains (Dopson et al., 2008). Both *Metallosphaera* strains solubilize metals by attaching to their respective substrates as well as by regenerating Fe^2+^ to Fe^3+^ that is vital for chemical attack on minerals (Bromfield et al., 2011; Kölbl et al., 2017). While the growth of *S. metallicus* is inhibited by a high amount of surrounding Fe^2+^ ions (Shiers et al., 2013), proliferation on a high Fe^3+^ content (as in BOF-dust) does not seem to have significant impact on cellular propagation since *S. metallicus* cells leach metals by attaching to a mineral surface as well as by providing Fe^3+^ ions as a lixiviant (Gautier et al., 2008). Whereas *S. metallicus* grows in an obligate chemolithoautotrophic mode, its relative *S. acidocaldarius* proliferates heterotrophically (Wheaton et al., 2015). This might explain the faster generation time of *S. acidocaldarius* in course of cultivation. In this study, *S. acidocaldarius* outperformed *S. metallicus* in the growth and bioleaching of most elements except for Fe. Contradictory experimental data on lifestyle and substrate utilization (autotrophy vs. heterotrophy; oxidation of S^0^) of *S. acidocaldarius* (Dworkin et al., 2006) need to be clarified by dedicated experiments in order to determine bioleaching mechanism(s). *A. manzaensis* is a facultative autotroph capable of utilizing Fe^3+^ as electron acceptor under anaerobic circumstances in contrast to the closely related *A. brierleyi* species (Yoshida et al., 2006). However, no data exist on how a high concentration of surrounding Fe^3+^ ions can influence the growth rate of *A. manzaensis*. While Fe^3+^ ions are a vital oxidizing agent in autotrophic bioleaching processes, the generation of protons by acidophilic microorganisms further supports bioleaching and keeps Fe^3+^ ions in solution (Donati and Sand, 2007). Both heterotrophs (e.g., fungi) and autotrophs (e.g., thermoacidophilic iron-oxidizing archaea) are capable of solubilizing metals from ores and minerals using different strategies and bioleaching agents (Schippers et al., 2013). In this study, all cultivated archaeal strains are chemolithotrophs (except *S. acidocaldarius*) that use a variety of inorganic energy sources, which are in turn closely intertwined with their bioleaching abilities (Donati and Sand, 2007). Chemolithotrophic production of sulfuric acid (protons) by sulfur-oxidizing thermoacidophilic archaea (*Metallosphaera* spp*., Acidianus* spp*., S. metallicus*) might additionally support efficient metal solubilization from BOF-dust grains by maintaining a low pH during bioleaching operations.

All six thermoacidophilic archaea were able to efficiently solubilize various metals from BOF-dust grains due to their inherent bioleaching capacities. Several enzymes involved in bioleaching processes have been identified among the Sulfolobales order. *A. manzaensis* genome encodes several enzymes involved in sulfur and RISCs oxidation, including a sulfur oxygenase-reductase, thiosulfate:quinone oxidoreductase and a tetrathionate hydrolase (Ma et al., 2017). So far, no genes for iron redox chemistry in *A. manzaensis* have been annotated, which makes future biochemical characterization on genes responsible for bioleaching capacity necessary. The *fox* gene cluster responsible for iron oxidation was identified in *S. metallicus* (Bathe and Norris, 2007) and *M. sedula*, as well as the genetic presence of rusticyanin homologs (Rus) for iron oxidation and tetrathionate hydrolase (TetH) for sulfur oxidation, appear to distinguish microbes able to perform bioleaching from non-bioleaching microbes (Auernik et al., 2008). Bioleaching (and/or biomining) capacities of *M. sedula, M. hakonensis, A. brierleyi, S. metallicus*, and *S. acidocaldarius* have been examined extensively compared to *A. manzaensis*. Comparative ICP-MS analysis of all thermoacidophiles shows superiority of *A. manzaensis* in leaching of certain strategically important elements from BOF-dust, such as Cu, Ni, Zn, Cr, Cd, and Mn (Figure 2). Future experiments to determine iron redox (bio)chemistry in *A. manzaensis* need to be carried out to fully understand its bioleaching potential.

Refinement of the current BOF-bioleaching operation included BOF-dust load, pH, and slight temperature shifts. For most elements (Fe, Zn, Mn, Al, Cu, Ni, Cr, Cd, Co, V, Sr, Ba, Mg), a higher dust load (Figure 3A) equals higher amounts leached into solution. In contrast, elements such as Ti, Pb, Mo, and Al might have formed insoluble precipitates under higher dust load conditions since they seem to be underrepresented in the analyzed bioleachate solution (Figure 3A). Furthermore, a decrease of operation pH from 2.5 to 1.5 increased Cr, Fe, and Ni bioleaching significantly compared to a less acidic pH (Figure 3B). On the contrary, Zn, Mn, Co, Cd, and Cu bioleaching was enhanced at higher pH (2.5). Similar results for *A. manzaensis* copper bioleaching at different pH-values have been shown by previous experiments, where Cu dissolution increased with a less acidic pH, whereas Fe concentration dropped by pH increase which is in concordance with own measurements (Liang et al., 2014; Liu et al., 2016a). In classic metal sulfide bioleaching, Fe precipitation is usually accounted to the formation of a passivating layer (usually by jarosite), which slows down overall mineral dissolution (Klauber, 2008). Excessive implementation of mesophilic microorganisms such as *Acidithiobacillus* spp. and *Leptospirillum* spp. in commercial bioleaching operations of metal sulfides (pyrite, chalcopyrite) shows that biological leaching processes decrease with time and eventually cease due to the formation of a polysulfide (Cu_4_Fe_2_S_9_) passivation layer on the mineral surface (Hackl et al., 1995; Zhao et al., 2019). Thermoacidophilic archaea such as *Acidianus* spp., *Sulfolobus* spp., and *Metallosphaera* spp. are able to significantly accelerate mineral sulfide dissolution, cope with high overall acidity and temperatures (Clark and Norris, 1996; Gericke and Pinches, 1999) and can promote a more efficient dissolution of BOF-dust grains by solubilizing metals into the leachate solution (Figure 2). The formation of unwanted or inhibiting bioleaching by-products formed by these processes needs to be investigated in further studies in order to enhance industrial implementation. Slight changes in bioleaching operation temperature (62°C/64°C/67°C) did not reveal any significant solubilization events; for future investigation, further temperature shifts should be examined in depth.

*A. manzaensis* was the most efficient strain among all other tested Sulfolobales spp. capable of chemolithotrophic growth on BOF-dust. *A. manzaensis* broke down the chemical matrix of BOF-dust grains and actively solubilized various metals into the leachate solution. Our SEM observations show the successful colonization of metal-bearing waste particles by *A. manzaensis* and their subsequent biotransformation (e.g., etched grains surface, Figures 4C–H, Figure 5, Supplementary Figure S3). This is in concordance with previous findings of cooperative contact/non-contact bioleaching mechanisms of *A. manzaensis* (Zhang et al., 2019). Studies of chalcopyrite bioleaching with *A. manzaensis* cells also indicate corrosion and break down of the mineral matrix with simultaneous copper solubilization after several days of cultivation (He et al., 2009; Liu et al., 2016b). Furthermore, the formation of a jarosite (KFe_3_(SO_4_)_2_(OH)_6_) passivation layer by *A. manzaensis* was reported from chalcopyrite bioleaching operations (Nie et al., 2019), indicating the formation of by-products during leaching processes. In our study, we observed gypsum crystals (CaSO_4_ × 2H_2_O) after approximately 7 days of cultivation (Figure 8), which might be accounted for both microbial and chemical-induced processes during cultivation. A high calcium content in the BOF-dust (Supplementary Table S1), as well as the involvement of sulfur derived from the same product, can account for abiotic calcium sulfate formation once these elements are intensively released by *A. manzaensis* into the leachate solution. Similar to calcium sulfate, nickel and manganese sulfate formation in the leachate solution has been previously reported for *M. sedula* cultivated on multimetallic extraterrestrial material (Milojevic et al., 2019b). In this case, associated with the potential for asteroid biomining operations, nickel sulfate was proposed as a metabolic biosignature resulting from the microbial leaching activity of meteorite minerals (Milojevic et al., 2019b). In light of terrestrial industrial operations, gypsum is a naturally occurring mineral and is used in various industrial settings. Usually, gypsum is formed as a by-product of sulfide oxidation to produce sulfuric acid and is used excessively in the construction industry (Lutz et al., 2012). In contrast, a study conducted by Vogel et al. shows that biofilms of cyanobacteria and *Bacteroidetes* species are actively involved in gypsum sedimentation implicating biological involvement in its formation (Vogel et al., 2009). Despite the possibility of biological involvement, a low pH favors the growth of gypsum crystals in inorganic systems (Liu et al., 2015). Furthermore, substrates rich in Fe, Ca, Mg, or Ba combined with continuous CO_2_ input in the bioreactor might precipitate as a stable red gypsum mineral through CO_2_ mineral carbonation (Rahmani et al., 2016; Rahmani, 2018, 2020). As the origin of gypsum crystals in this study is not resolved, more studies on its influence on bioleaching efficiency and its possible application as a useful by-product need to be executed.

First insights into laboratory-scale thermoacidophilic bioleaching associated with industrial steelmaking waste products are provided in this study. Further genetic analysis and biochemical characterization of metal-oxidizing molecular machinery of *A. manzaensis* need to be carried out to optimize this strain for future operations. A thorough screening of operation parameters in frames of scale-up processes is highly necessary to implement this technology for upcoming circular processes in order to efficiently recycle/re-feed valuable metals into industrial systems. In addition to microbial analysis on a nanometer-scale, metalliferous waste products need to be sufficiently characterized in order to assess bioleaching efficiency of each tested microorganism.

## Conclusion

Six thermoacidophilic archaeal strains (*M. sedula, M. hakonensis, S. acidocaldarius, S. metallicus, A. brierleyi*, and *A. manzaensis*) were capable of growing when supplemented with multimetallic BOF-dust derived from a steel production plant. Furthermore, each species was able to leach a set of elements from BOF-dust grains; however, *A. manzaensis* showed the highest potential in growth and bioleaching of economically relevant elements. Electron microscopy-assisted analysis of BOF-dust grains processed by *A. manzaensis* reveals a breakdown of the dust-grains mineral matrix in course of cultivation. Additionally, the formation of gypsum crystals was observed during the cultivation process. The obtained results emphasize the utilization of thermoacidophilic archaea for future (industrial) bioleaching operations and the need to conduct more in-depth studies to optimize these processes. So far, bioleaching mechanisms have been described extensively for a handful of acidophilic bacteria (overview by Donati and Sand, 2007). To exploit the full potential of thermoacidophilic archaea, more in-depth studies need to be carried out for the future efficient bioleaching operations and for the development of environmental-friendly circular economies. Further investigations should focus on resolving the microbial-mineral interfaces of the selected thermoacidophiles grown on waste product materials down to nanometer scale. This will help understand the mechanisms of microbial interactions with metal-bearing waste product material and see the relevant cell compartments involved in the bioleaching process.

## Data Availability Statement

The original contributions presented in the study are included in the article/Supplementary Material, further inquiries can be directed to the corresponding author.

## Author Contributions

DK, AM, MA, MB, HS, and TM performed experiments. DK, AM, and TM planned and executed the cultivation of thermoacidophilic archaea, as well as performance and interpretation, of SEM-EDS experiments. MB performed ICP-MS analyses. MA and TM performed and analyzed STEM-EDS results. All authors made substantial contributions to the acquisition, analysis, and interpretation of the data described in this study. All authors critically reviewed the manuscript and approved the final version of it.

## Conflict of Interest

GK, HS, and DW were employed by the company voestalpine Stahl Donawitz GmbH, Leoben, Austria.

The remaining authors declare that the research was conducted in the absence of any commercial or financial relationships that could be construed as a potential conflict of interest.

## Publisher's Note

All claims expressed in this article are solely those of the authors and do not necessarily represent those of their affiliated organizations, or those of the publisher, the editors and the reviewers. Any product that may be evaluated in this article, or claim that may be made by its manufacturer, is not guaranteed or endorsed by the publisher.
