# Supplementary material for: Thermoacidophilic Bioleaching of Industrial Metallic Steel Waste Product
Source: Front Microbiol. 2022 Apr 13;13:864411. doi: 10.3389/fmicb.2022.864411 (PMC9043896; doi:10.3389/fmicb.2022.864411)
Supplement: Supplementary file 1 [file Data_Sheet_1.PDF]

Supplementary Material

Supplementary Figures

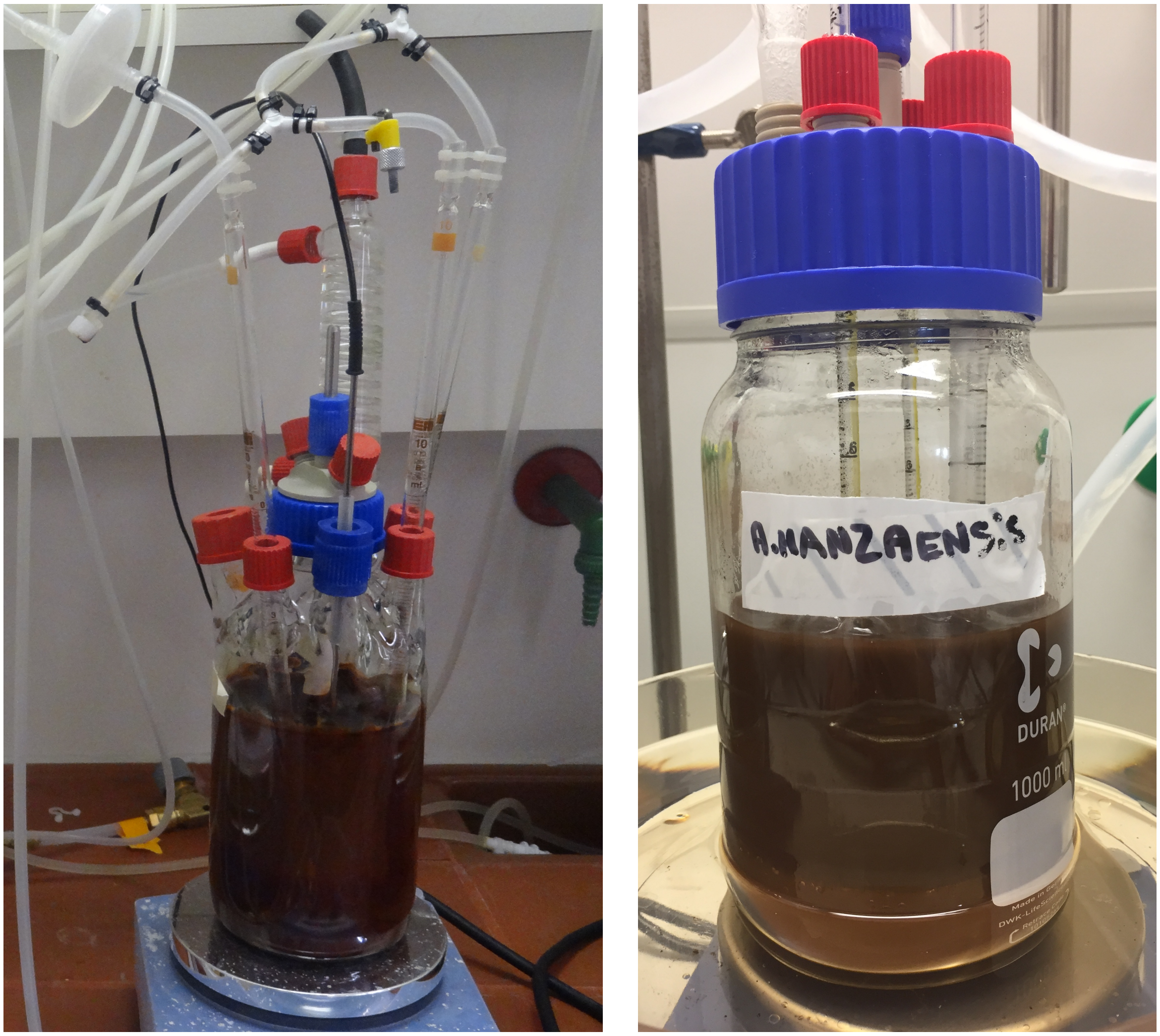

Supplementary Figure 1. Fermentation set up for cultivation of thermoacidophiles in 1L glassblower modified Schott-bottle bioreactors.

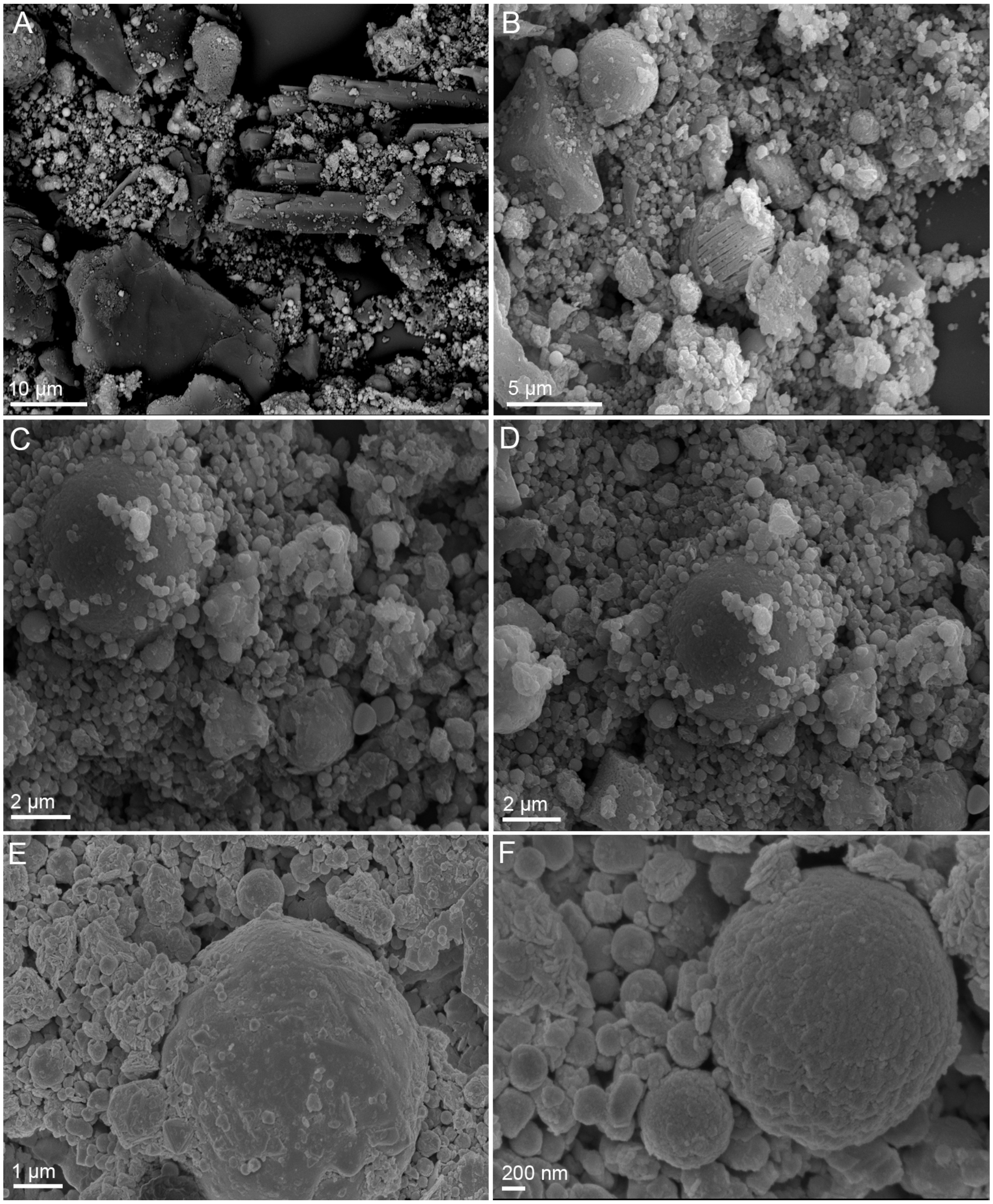

Supplementary Figure 2. Additional scanning electron microscopy (SEM) images of BOF-dust particles and cells of *A. manzaensis*.

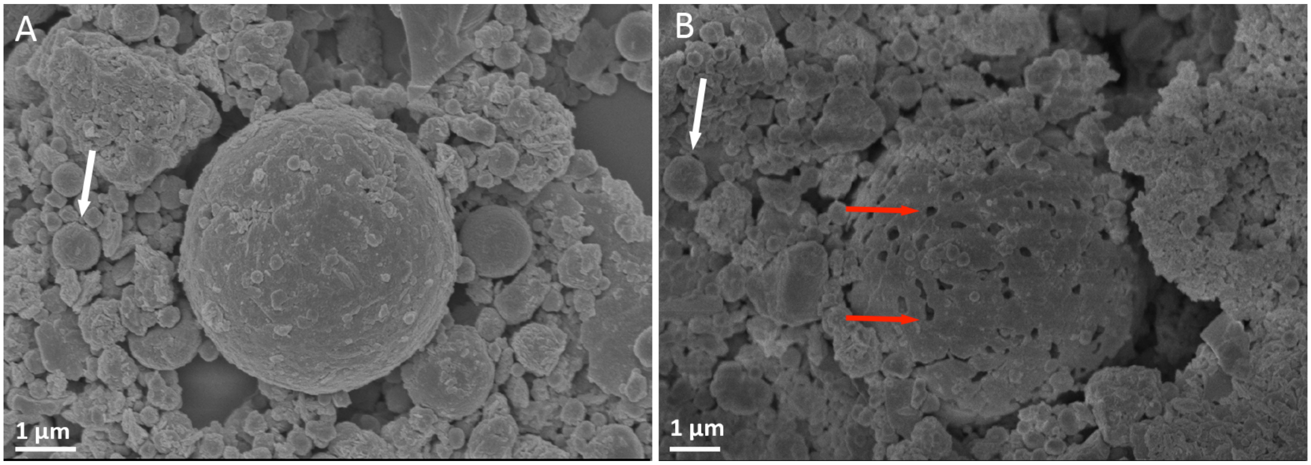

Supplementary Figure 3. Additional scanning electron microscopy (SEM) images showing the microbially etched surface of grains of BOF-dust bioprocessed by *A. manzaensis*. The microbially etched pits are depicted by the red arrows. The cells are around 1 µm size and are depicted by the white arrows.

Table 1. Wavelength dispersive X-ray fluorescence analysis (WD-XRF) of BOF-dust used in the study.

Parameter	Wt%
Fe_2O_3	**39.5**
CaO	**15.3**
MgO	**14.9**
SiO_2	**7.6**
K_2O	**3.3**
ZnO	**3.0**
MnO	**2.0**
SO_3	**1.7**
Na_2O	**1.5**
Al_2O_3	**1.4**
Cl	**0.9**
PbO	**0.36**
P_2O_5	**0.17**
TiO_2	**0.09**
Cr_2O_3	**0.07**
SrO	**0.02**
Co_3O_4	**0.02**
V_2O_5	**<0.01**
NiO	**<0.01**
MoO_3	**<0.01**
CdO	**<0.01**
BaO	**<0.01**
